# Study of Microstructural Morphology of Ti-6Al-4V Alloy by Crystallographic Analysis and Phase Field Simulation

**DOI:** 10.3390/ma15155325

**Published:** 2022-08-02

**Authors:** Hao Xiang, Wim Van Paepegem, Leo A. I. Kestens

**Affiliations:** 1 Department of Electromechanical, Systems and Metal Engineering, Faculty of Engineering and Architecture, Ghent University, Tech-Lane Ghent Science Park-Campus A, Technologiepark Zwijnaarde 46, B-9052 Ghent, Belgium; 2 Department of Materials, Textiles and Chemical Engineering (MaTCh), Faculty of Engineering and Architecture, Ghent University, Tech-Lane Ghent Science Park-Campus A, Technologiepark Zwijnaarde 46, B-9052 Ghent, Belgium; wim.vanpaepegem@ugent.be

**Keywords:** Ti-6Al-4V, martensitic transformation, phase field simulation, twins, Phenomenological Theory of Martensitic Transformation

## Abstract

Formation of a habit plane during martensitic transformation is related to an invariant plane strain transformation, which involves dislocation glide and twins. In the current work, the Phenomenological Theory of Martensitic Transformation (PTMT) is employed to study the crystallographic features while the phase field simulation is used to study the microstructure evolution for martensitic transformation of Ti-6Al-4V alloy. Results show that mechanical constraints play a key role in the microstructure evolution. It is shown that a twinned structure with very small twinned variants is geometrically difficult to form due to the lattice parameters of Ti-6Al-4V alloy. It is concluded that the predicted habit plane from the PTMT is consistent with results of the micro-elastic theory. The formation of a triangular morphology is favored geometrically and elastically.

## 1. Introduction

Ti-6Al-4V alloy, known as TC4, is widely applied in different fields due to its excellent mechanical properties, corrosion resistance and superior biocompatibility [1,2,3,4,5,6,7,8,9]. Complex microstructures of Ti-6Al-4V alloy may be observed as a result of different manufacturing procedures due to the phase transformation from the high-temperature β phase (BCC) to the low-temperature α phase (HCP). In order to improve the alloy’s mechanical performance and industrial applications, it is important to control the manufacturing procedure by understanding the mechanism and microstructure evolution during martensitic transformation.

During martensitic transformation, dislocation glide and/or twins are involved to accommodate the shear strain and produce the habit plane. Yang et al. [10] used High-Resolution Transmission electron microscopy (HRTEM) to observe partial dislocations at the atomic scale during phase transformation in AISI 304 austenitic stainless steel. Liu et al. [11] confirmed the interaction between dislocations and transformed $\alpha^{\prime }$ martensite and concluded that dislocations are piling up at grain boundaries to induce the nucleation of new $\alpha^{\prime }$ martensite. In terms of twinned microstructures, Bhattacharya proposed several different twins with compatibility functions [12]. Except for the different twins classified by the twinning elements, including the compound twin, type I and II twins [13], the crossing twins were considered, involving four variants that have been observed and predicted in NiTi and Ti2448 alloy [14,15,16,17]. For the Ti-6Al-4V alloy it was reported that twinned microstructures are attributed to the plastic deformation, which can release significantly the stress concentration around micro-crack [18,19], but not much research is reported about the transformation twins within Ti-6Al-4V alloy [20,21].

In addition to the direct observation of microstructures, many different modeling methods have been proposed to explain the nature and features of martensitic transformation. One simple, geometrical and generally accepted crystallographic theory is the Phenomenological Theory of Martensitic Transformation, which was proposed by Greninger-Troiano [22] and developed by Bowles and Mackenzie (B-M theory) [23,24] and Wechsler, Lieberman and Read (W-L-R theory) [25,26], respectively. Based on the lattice parameters, Lieberman [27] and Sun et al. [28] successfully captured important microstructural features of AuCd alloy and Ti-Nb based shape memory alloys, respectively. Another geometrical method is the topological model (TM) constructed by Pond and Hirth [29]. Unlike the PTMT theory, the habit plane and orientation relationship between parent and product phase are predicted based on the interfacial defects, but without High Resolution TEM the crystallographic geometry of interfacial disconnections is difficult to confirm [30,31]. Due to the simple calculation and highly accurate physical results from the PTMT theory, it has been employed widely to obtain the crystallographic features of alloys during martensitic transformation [21,32,33,34,35,36].

For the martensitic transformation, in addition to the predicted habit plane and shear deformation, it is also important to simulate the microstructure evolution under different conditions. One powerful simulation method is phase field modelling based on the thermodynamic and micro-elastic kinetic potential, which was proposed by Cahn and Allen, and developed by Khachaturyan and Wang [37,38,39,40]. Phase field models are applied widely to simulate microstructure evolution for different alloys and study the effect of chemical composition, internal or external mechanical or magnetic loads on microstructure and fracture [41,42,43,44,45]. Through phase field simulation, Shi and Qiu et al. [46,47,48,49] have studied the effect of external loading on variant selection mechanisms and the self-accommodation phenomena of Ti-6Al-4V alloy. However, there are few reports [20,21] about the role of twins in the transformation strain accommodation in Ti-6Al-4V alloy. In the current work, the Phenomenological Theory of Martensitic Transformation is employed to analyze the crystallographic features of twinned microstructure and phase field modelling is used to simulate the microstructure evolution and compare the results with experiments.

## 2. Crystallographic Analysis by PTMT

### 2.1. Transformation Matrix

**Figure 1:**
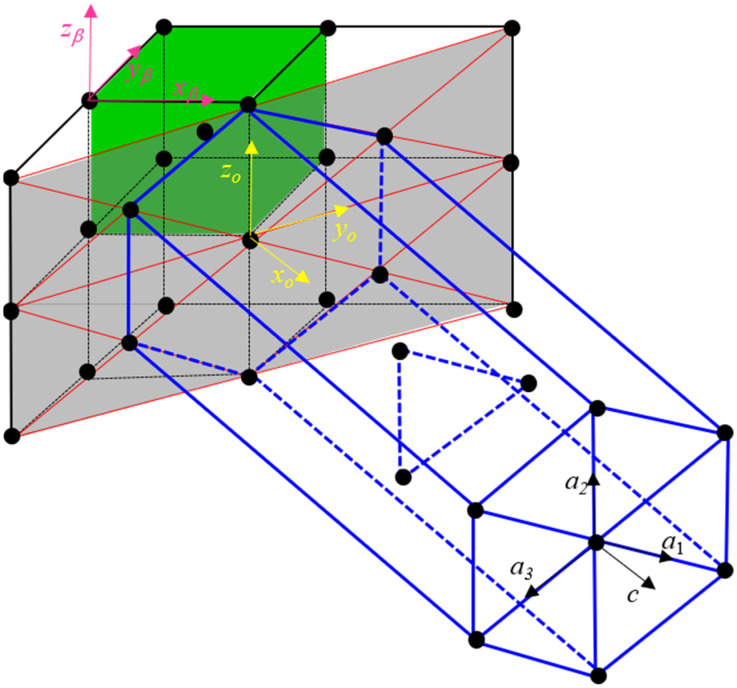
Crystal lattice of parent BCC and product HCP phase, and orthogonal lattice correspondence between BCC and HCP phase.

Theoretically, unlike diffusion transformations, martensitic transformations (also known as displacive transformations) involve short-range atomic displacements, which result in a change of crystal structure with minimum transformation elastic strain energy. Based on the Burgers orientation correspondence [50], for the martensitic transformation of Ti-6Al-4V alloy, the lattice orthohexagonal reference system between BCC and HCP cells is shown in Figure 1, and it corresponds to:

$$x_{o}\left|\left|\left[1\;0\;0\right]\right|\right|{\left[1\;\bar{1}\;0\right]}_{\beta }||{\left[0\;0\;0\;1\right]}_{\alpha };\;y_{o}\left|\left|\left[0\;1\;0\right]\right|\right|{\left[1\;1\;0\right]}_{\beta }||{\left[1\;0\;\bar{1}\;0\right]}_{\alpha };\;z_{o}\left|\left|\left[0\;0\;1\right]\right|\right|{\left[0\;0\;1\right]}_{\beta }||{\left[\bar{1}\;2\;\bar{1}\;0\right]}_{\alpha };$$

Within such a lattice correspondence system, the transformation matrix in the $n_{o}$= ($x_{o}\;y_{o}\;z_{o}$) orthogonal reference frame is:

(1) $$U_{o}=\left[\begin{array}{ccc} \frac{c_{\alpha }}{\sqrt{2}a_{\beta }} & 0 & 0\\ 0 & \frac{\sqrt{3}a_{\alpha }}{\sqrt{2}a_{\beta }} & 0\\ 0 & 0 & \frac{a_{\alpha }}{a_{\beta }} \end{array}\right]$$

where the lattice parameters of α and β phase are: $a_{\alpha }$ = 0.29511 nm, $c_{\alpha }$ = 0.46843 nm and $a_{\beta }$ = 0.33065 nm, respectively [51]. Then the transformation matrix of one variant shown in the parent β coordinate system is:

(2) $$U_{\beta }=\left[\begin{array}{ccc} 1.0474 & 0.0457 & 0\\ 0.0457 & 1.0474 & 0\\ 0 & 0 & 0.8925 \end{array}\right]$$

Theoretically, the atomic shuffle is necessary for phase transformation between BCC and HCP crystallographic structures through dislocation glide [52,53,54]. Therefore, in the current work, without considering the internal shuffle, only six correspondence variants from a single parent beta grain are produced (in order to distinguish from the 12 crystallographic variants in which the dislocation glide is under consideration, the phrase ‘correspondence variant’ is used, and ‘variant’ refers to the correspondence variant without specifying the ‘crystallographic variants’). The transformation matrices for all six variants can be calculated with the cubic symmetry operators and listed below:

(3) $$U_{1}=\left[\begin{array}{ccc} 0.8925 & 0 & 0\\ 0 & \;1.0474 & -0.0457\\ 0 & -0.0457 & \;1.0474 \end{array}\right],\;U_{2}=\left[\begin{array}{ccc} 0.8925 & 0 & 0\\ 0 & 1.0474 & 0.0457\\ 0 & 0.0457 & 1.0474 \end{array}\right],\;U_{3}=\left[\begin{array}{ccc} 1.0474 & 0 & -0.0457\\ 0 & 0.8925 & 0\\ -0.0457 & 0 & 1.0474 \end{array}\right],\;U_{4}=\left[\begin{array}{ccc} 1.0474 & 0 & 0.0457\\ 0 & 0.8925 & 0\\ 0.0457 & 0 & 1.0474 \end{array}\right],\;U_{5}=\left[-\begin{array}{ccc} 1.0474 & -0.0457 & 0\\ 0.0457 & 1.0474 & 0\\ 0 & 0 & 0.8925 \end{array}\right],\;U_{6}=\left[\begin{array}{ccc} 1.0474 & 0.0457 & 0\\ 0.0457 & 1.0474 & 0\\ 0 & 0 & 0.8925 \end{array}\right],\;$$

### 2.2. Habit Plane between Single Variant and Matrix

In order to form an invariant plane between a single variant and the matrix, the kinetic compatibility function is considered, based on Bhattacharya [12], and can be written as:

(4) $$R_{i}U_{i}-I=b⊗m$$

where $R_{i}$ is a rigid body rotation of variant *i* with respect to parent phase, $U_{i}$ is the transformation matrix of variant *i* in Equation (3), ***I*** is the unit matrix, **b** and **m** are shape strain and habit plane normal, respectively and ⊗ represents the dyadic product of two tensors. From Ball and James’ work [55], we know that, only if the eigenvalues of $U_{i}^{2}$ meet the condition $\lambda_{1}<\lambda_{2}=1<\lambda_{3}$, does Equation (4) have a solution for the invariant plane. In the present study, the eigenvalues of $U_{1}^{2}$ are 0.7966, 1.0034 and 1.1949, respectively. Therefore, the transformation matrix from a single variant can induce the formation of an invariant plane between variant and matrix without involving the lattice invariant shear. With the subroutine of Hane and Shield [56], solutions of Equation (4) for all 6 variants are obtained and listed in Table 1. Even though the interface between the parent β phase and product α phase may partially lose its coherency, the observed {3 3 4} habit plane normal [57] has been widely accepted. Based on Srivastava’s work [57], the predicted habit plane normal from the PTMT calculation exhibits a small angular deviation of 2.3° with respect to observed {3 3 4} habit plane normal, which is generally acceptable within an assumed tolerance of 5°. It can be concluded that the prediction of the habit plane normal for a single variant agrees well with experimental results.

### 2.3. Invariant Plane in Twinned Microstructure

Aside from the habit plane between a single variant and parent matrix, the invariant plane can be formed within two twinned variants, which is governed by Equation (5). With two twinned variants, the invariant plane between twinned variants and matrix can be solved with the kinetic compatibility Equation (6):

(5) $$R_{ij}U_{i}-U_{j}=a⊗n$$

(6) $$R[\lambda R_{ij}U_{i}+\left(1-\lambda \right)U_{j}]=I+b⊗m$$

where $R_{ij}$ is a rigid body rotation of variant *i* with respect to variant *j*, a and n are a shear vector and twin plane normal, respectively: λ is the volume fraction of the twinned variant *i*; ***R*** is a rigid body rotation of the twinned region with respect to the parent phase. The transformation strain of the twinned region for Equation (6) is taken as the average strain of the two twinned variants. In terms of solutions of twinned region for all combinations with six variants, there are six compound twins, 12 type I and 12 type II twins. The results are listed in Table 2, Table 3 and Table 4. It should be pointed out that the predicted twin plane of compound and type I are already observed [58]. The twin planes of {1 0 0} and {1 1 0} are transformed into {$\bar{2}\;1\;1\;0$} and {0 0 0 1} plane in the hexagonal cell, respectively. However, the angular deviation of the predicted twin plane of type II from the frequently observed {1 1 2} twin is 16.82°, whereby the {1 1 2} β phase twin plane is transformed into a {$\bar{1}\;\bar{1}\;2\;3$} twin plane in the hexagonal cell [58]. With the solutions of $R_{ij}$, the volume fraction of twinned variants, and invariant plane between twinned region and parent β phase can be predicted based on Equation (6). Here the combination of variants 2 and 5 is taken as an example, and the predicted results are listed in Table 5. It can be seen that for the twinned region, the volume fraction of the minor part is very small, with ~4%, which is consistent with the observed TEM morphology of Ti-6Al-4V alloy [59,60,61]. The small volume fraction within twinned region would be the reason that the transformation twin microstructure is difficult to observe for Ti-6Al-4V alloy.

### 2.4. Orientation Relationship

For the twinned region with two variants, the total macroscopic distortion ***E*** is:

(7) $$E=R[\lambda R_{ij}U_{i}+\left(1-\lambda \right)U_{j}]=\lambda RR_{ij}U_{i}+\left(1-\lambda \right)RU_{j}=\lambda E_{i}+\left(1-\lambda \right)E_{j}$$

where shape deformation $E_{i}$ and $E_{j}$ originate from variant *i* and *j*, respectively. Therefore, any vector $v_{\beta }$ in the parent β phase will be transformed into $v_{\alpha }$ in the product α phase with total shape deformation and can be separated into two parts corresponding to each twinned variant, cf. Equation (8). As a result, the orientation relationship between parent phase and each twinned variant depends on the distortions to which each twinned variant is subjected separately, i.e., it depends on $E_{i}$ and $E_{j}$.

(8) $$v_{\alpha }=Ev_{\beta }=\lambda v_{\alpha i}+\left(1-\lambda \right)v_{\alpha j}=\lambda E_{i}v_{\beta }+\left(1-\lambda \right)E_{j}v_{\beta }$$

In order to compare the predicted orientation relationship with experimental results, the type I twin with combinations of variant 1/3, 2/5 and 4/6 are selected in the current work, and the {0 0 0 1}, {1 0 $\bar{1}$ 0} planes and <1 1 $\bar{2}$ 0> directions are checked.

The (0 0 0 1) and (1 0 $\bar{1}$ 0) planes in the hexagonal unit cell correspond to (1 0 0) and (0 1 0) in the orthogonal $n_{o}$ coordinate system; thus, the (1 0 0) plane from variant 1 and 3 back-transformed to the parent β phase by the distortion tensors $E_{1}^{\prime }$ and $E_{3}^{\prime }$ are (−0.0999 −0.7030 0.7042) and (−0.7269 −0.0743 0.6827), respectively. The angular deviation of the predicted back-transformed planes from (0 $\bar{1}$ 1) and ($\bar{1}$ 0 1) are 5.7329° and 4.6233°. The angular deviations of compared (0 1 0) in orthogonal $n_{o}$ coordinate system for variant 1 and 3 are 0.0715° and 2.2665° from (0 1 1) and (1 0 1) plane in parent beta phase, respectively. The predicted orientation relationship for the planes can be written as:

$${\left(0\;\bar{1}\;1\right)}_{\beta }5.7329{}^{\circ}\;\mathrm{from}\;\left(0\;0\;0\;1\right){\;}_{\alpha }^{1},\;{\left(\bar{1}\;0\;1\right)}_{\beta }4.6233{}^{\circ}\;\mathrm{from}\;\left(0\;0\;0\;1\right){\;}_{\alpha }^{3},\;{\left(0\;1\;1\right)}_{\beta }0.0715{}^{\circ}\;\mathrm{from}\;\left(1\;0\;\bar{1}\;0\right){\;}_{\alpha }^{1},\;{\left(1\;0\;1\right)}_{\beta }2.2665{}^{\circ}\;\mathrm{from}\;\left(1\;0\;\bar{1}\;0\right){\;}_{\alpha }^{3},$$

Similar to the calculation for predicting transformed planes, the orientation relationship for crystallographic directions can be obtained. The selected [1 1 $\bar{2}$ 0] direction from hexagonal cell has the coordinates [0 0.8660 0.5000] in the orthogonal $\;n_{o}$ coordinate system by applying the hexagonal lattice parameters. These correspond to the unit vectors [$\bar{0.4982}\;0.6479\;0.5762]$ and [0.5571 0.5175 0.6495] in the parent β phase for variant 1 and 3, the angular deviations of these predicted directions are 6.0797° and 5.4979° from [$\bar{1}\;1\;1$] and [1 1 1], respectively. The results are written as:

$${\left[\bar{1}\;1\;1\right]}_{\beta }6.0797{}^{\circ}\;\mathrm{from}\;{\left[1\;1\;\bar{2}\;0\right]}_{\alpha }^{1},\;{\left[1\;1\;1\right]}_{\beta }5.4979{}^{\circ}\;\mathrm{from}\;{\left[1\;1\;\bar{2}\;0\right]}_{\alpha }^{3},$$

The predicted orientation relationship for all six variants with selected planes and direction are listed in Table 6. It should be noticed that the predicted orientation relationship of certain variants can be obtained from different twinned regions, such as the orientation relationship of variant 1 can be captured from twinned variants 1/3 and 1/4. Hence, the results for variant 1 would exhibit minor differences due to different volume fractions of different twinned variants. The results, shown in Table 6, are based on the minimum angular deviations.

### 2.5. Cross-Twin Structure

Bhattacharya [12] mentioned that, for some alloys, a special crossing twinned microstructure would be formed with four transformation variants during phase transformation. The compatibility functions for such crossing twins are:

(9a) $$R_{ji}U_{j}-U_{i}=a_{1}⊗n_{1};\;$$

(9b) $$R_{kj}U_{k}-U_{j}=a_{2}⊗n_{2};\;$$

(9c) $$R_{lk}U_{l}-U_{k}=a_{3}⊗n_{3};\;$$

(9d) $$R_{il}U_{i}-U_{l}=a_{4}⊗n_{4};\;$$

(9e) $$R_{ji}R_{kj}R_{lk}R_{il}=I;\;$$

(9f) $$n_{1},\;n_{2},\;n_{3}\;\mathrm{and}\;n_{4}\;\mathrm{in}\;a\;\mathrm{plane}\;$$

The meaning of the quantities of Equation (9) are the same as in Equation (5). Equations (9a)–(9d) generate the interfacial invariant planes between two variants. Equation (9e) guarantees that a quadruple junction point is formed without dislocations along interaction lines of variants and Equation (9f) makes sure that all four twin planes meet along a line. The solutions of the crossing twin will be discussed later.

## 3. Phase Field Simulation

Given a set of lattice parameters, the Phenomenological Theory of Martensitic Transformation above can capture important crystallographic features during martensitic transformation through geometrical analysis. The habit plane between variants and matrix can also be predicted by phase field simulation based on the micro-elastic (KS) theory [37,38,39,40]. Phase field modelling is usually employed to study microstructural evolution during martensitic transformation with different conditions [16,17,39,40]. In the present work, the stress-free transformation strain (SFTS) during β to α martensitic transformation is set at input to simulate the microstructure evolution for all six variants by time-dependent Ginzburg–Landau (TDGL) equation.

### 3.1. Stress-Free Transformation Strain

During martensitic transformation, the change of lattice structure leads to transformation deformation. The SFTS can be calculated with following equation [16,17]:

(10) $$\varepsilon_{ij}^{00}\left(p\right)=\frac{U_{p}^{\prime }U_{p}-I}{2}\;(p=1\sim 6)\;$$

In Equation (10) **I** refers the unit matrix, $U_{p}$ is the transformation matrix and $U_{p}^{\prime }$ its transpose.

### 3.2. Free Energy Formulation

In the current study, six non-conserved fields $\eta_{p}$ are introduced to present six different correspondence variants, where $\eta_{i}\left(r\right)\;$= 1 stands for variant *i* in position r, while $\eta_{i=1\sim 6}\left(r\right)\;$= 0 stands for β matrix. Here the atomic shuffle is not under consideration. Therefore, the local chemical free Gibbs energy can be expressed in a Landau-type polynomial [62] as:

(11) $$F^{ch}=\frac{1}{V_{m}}\left[\frac{A}{2}\sum_{p=1}^{6}\eta_{p}^{2}-\frac{B}{3}\sum_{p=1}^{6}\eta_{p}^{3}+\frac{C}{4}{\left(\sum_{p=1}^{6}\eta_{p}^{2}\right)}^{2}\right]$$

where A = 32$\Delta G^{*}$, B = 3A + 12$\Delta G_{m}$ and C = 2A + 12$\Delta G_{m}$ are expansion coefficients, $\Delta G_{m}$ is the driving force, which is the difference of Gibbs free energy between the β and α phases in equilibrium status, $\Delta G^{*}$ is the Gibbs energy barrier that opposes the martensitic transformation and $V_{m}$ is the molar volume.

The gradient energy in the interfacial region between α variants and β matrix in non-equilibrium conditions is given as:

(12) $$F^{grad}=\frac{1}{2}k\left|\frac{\partial \eta_{p}\left(r,t\right)}{\partial x_{i}}\right|\left|\frac{\partial \eta_{p}\left(r,t\right)}{\partial x_{j}}\right|$$

where k is the gradient energy coefficient under the assumption of isotropic interface between α variants and β matrix. When the simulation system reaches a stable status, the non-conserved structural parameter profiles remain constant in the local reference system attached to the interface, hence the Gibbs energy barrier $\Delta G^{*}$ and gradient energy coefficient k are related to interfacial energy γ by the following equations [62]:

(13) $$\Delta G^{*}=\frac{3\gamma V_{m}}{4\sqrt{2}\delta },\;k=\frac{3\gamma \delta }{2\sqrt{2}}$$

For a martensitic transformation, the local elastic strain energy is attributed to the change of crystallographic cells. Therefore, the local transformation elastic strain is assumed to be a linear superposition of SFTS with all local variants, given as:

(14) $$\varepsilon_{ij}\left(r\right)=\overset{6}{\underset{p=1}{\sum }}\eta_{p}\left(r\right)\varepsilon_{ij}^{00}\left(p\right)$$

With the local transformation elastic strain, the elastic strain energy of the simulated system can be formulated as:

(15) $$F^{el}=\frac{1}{2}\overset{6}{\underset{p=1}{\sum }}\oint^{\;}\frac{d^{3}k}{{\left(2\pi \right)}^{3}}B_{pq}\left(n\right){\left\{{\tilde{\eta }}_{p}\right\}}_{k}{\left\{{\tilde{\eta }}_{q}\right\}}_{k}^{*}$$

where the elastic strain energy density $B_{pq}$ in a clamped boundary condition is represented by [39,40]:

(16) $$B_{pq}\left(n\right)=\{\begin{array}{c} C_{ijkl}\varepsilon_{ij}^{00}\left(p\right)\varepsilon_{kl}^{00}\left(q\right)\;n=0\\ C_{ijkl}\varepsilon_{ij}^{00}\left(p\right)\varepsilon_{kl}^{00}\left(q\right)-n_{i}\sigma_{ij}^{00}\left(p\right)\Omega_{jk}\left(n\right)\sigma_{kl}^{00}\left(q\right)n_{l}\;n\neq 0 \end{array}$$

In Equations (15) and (16), $C_{ijkl}$ is the elastic stiffness tensor under the assumption that its components for α and β phases are the same and isotropic in a homogeneous simulation system, $\varepsilon_{kl}^{00}\left(p\right)$ is the SFTS of variant *p*, $\sigma_{kl}^{00}\left(p\right)=C_{ijkl}\varepsilon_{ij}^{00}\left(p\right)$ and $\Omega_{il}^{-1}\left(n\right)=C_{ijkl}n_{j}n_{k}$ is the inverse of the Green’s function in the reciprocal space, $n=\frac{k}{\left|k\right|}$ is a unit vector and k is a vector in the reciprocal space, ${\left\{{\tilde{\eta }}_{p}\right\}}_{k}$ is the Fourier transformation of $\eta_{p}\left(r\right)$, r is the position vector in real space and the asterisk stands for the complex conjugate.

As a result, the total free energy of system is given by:

(17) $$F=\int (F^{ch}+F^{grad})\;d^{3}r+\;F^{el}$$

### 3.3. Kinetic Equation

The Allen–Cahn equation is employed to describe the time dependent microstructure evolution during martensitic transformation:

(18) $$\frac{\partial \eta_{p}}{\partial t}=-M\frac{\partial F}{\partial \eta_{p}\left(r,t\right)}+\xi_{p}\left(r,t\right),\;\left(p=1\sim 6\right)$$

where *M* is the kinetic coefficient and $\xi_{p}\left(r,t\right)$ is the Langevin random noise term that describes the local thermal fluctuation of structural order parameters [63,64,65].

### 3.4. Simulation Parameters

In order to simplify calculations and save time, all simulation parameters are used in their reduced form as proposed by Chen et al. [66]:

(19) $$\tilde{F}=F/{\Delta G}_{m},\;\tilde{k}=k*V_{m}/\left(\Delta M*dx\right),\;\tilde{dt}=dt*\Delta M*{\Delta G}_{m}/{\left(dx\right)}^{2},\;\tilde{M}=M*{\left(dx\right)}^{2}/\left(\Delta M*V_{m}\right)$$

Based on Lindwall’s work [67], by setting the simulation temperature as 1073 K, and with equilibrium compositions of α phase and β matrix as Ti-10.25Al-3.21V and Ti-9.01Al-10.99 V (at%), the driving force ${\Delta G}_{m}$ can be obtained. All simulation parameters used in phase field modelling are listed in Table 7 below.

## 4. Simulation Results

In the current work, the phase field simulations are carried out with periodic boundary conditions in all three directions. No external load is applied. The Langevin random noises are introduced in the early simulation stage. Once enough nuclei have been formed, the noise term is turned off. With the SFTS as input, the Allen–Cahn function (Equation (18)) is solved by semi-implicit Fourier-spectral method in Fourier space [68].

### 4.1. Microstructure Evolution

**Figure 2:**
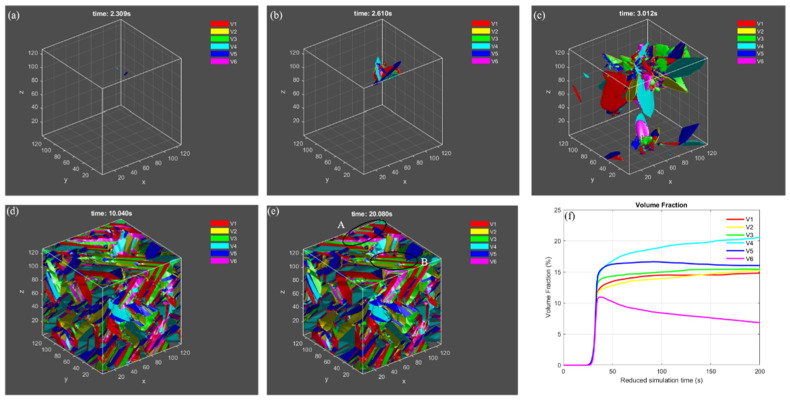
Microstructure evolution during martensitic transformation (**a**–**e**), and volume fraction of variants as a function of reduced simulation time (**f**).

**Figure 3:**
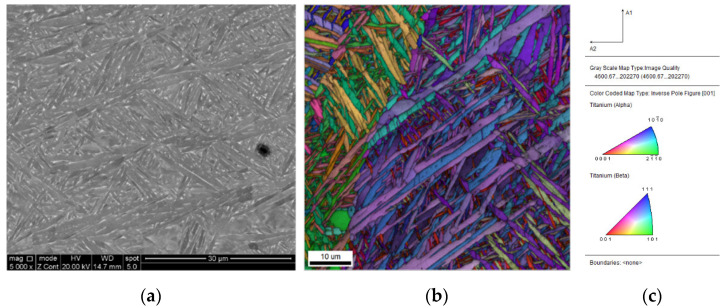
Microstructure observation: (**a**) Secondary electron SEM image; (**b**) EBSD scan color coded according to the inverse pole figure legend shown in (**c**) the details of EBSD observation are reported in Ref. [69].

Figure 2 shows the microstructure evolution and volume fraction of variants during martensitic transformation predicted by the phase field simulation, including nucleation, growth and twin formation. The β matrix in Figure 2a–e is set to be transparent, and the morphology of different α variants are outlined based on the condition that $\eta_{p}^{2}\left(r\right)\geq 0.5$ with different colors. As obtained by the PTMT above, a habit plane would be formed between a single α variant and β matrix. In Figure 2a, two variants 4 and 5 are nucleated in the early transformation stage. During growth they assume a plate shape with different directions, and the remaining variants nucleate and grow around the primary variant 4 and 5, which are induced by the interaction energy between variants, known as autocatalytic effect. A small triangular pyramidic structure is formed with 3 variants of 1, 4 and 5 (Figure 2b). While the martensitic transformation continues, different variants keep growing, impinge on each other (Figure 2c) and rearrange the morphology into a complex microstructure. Some small regions display twinned microstructure, such as area A and B in Figure 2e. Figure 2f shows the change-of-volume fraction for different variants. Most β matrices transform to product α variants but ~11% parent phased is still retained after 200s. Even though the quantitative comparison of microstructure between simulation and experiments is absent, the twinned microstructure and triangular morphology in current simulation agrees well with experimental observation on Selective Laser Melted (SLM) Ti-6Al-4V alloy in Figure 3. The microstructure evolution simulated by Phase- Field model can found in the Supplementary Materials.

### 4.2. Twinned Microstructure

**Figure 4:**
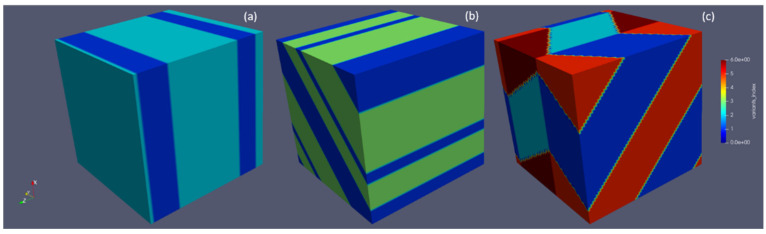
Twinned microstructure with different variant combinations: (**a**) compound twin; (**b**) Type I twin and (**c**) Type II twin.

As calculated by the PTMT, compound Type I and Type II twins would form with different variant combinations. In order to compare the predicted twins, several phase field simulations are employed for the formation of twins. It can be seen from Figure 4 that, with a small Langevin noise term, the autocatalytic effect would arrange the variants’ morphologies according to the minimum elastic energy path, leading to a twinned microstructure, which is also favored geometrically by PTMT.

## 5. Discussion

### 5.1. The Habit Plane between α Variants and β Matrix

With the specific lattice parameters of HCP and BCC crystallographic structure in Ti-6Al-4V alloy, the PTMT can predict most important crystallographic features with small angular deviations during martensitic transformation. Additionally, the orientation relationship between parent β phase and product α phase, the habit plane and microstructure evolution can also be captured by phase field modelling.

In the current study, the effect of external loading, chemical elements and temperature is not considered. Hence, the microstructure evolution is driven by the elastic strain energy from phase transformation. With the micro-elastic theoretical framework described in Part 4, the habit plane of a single variant can be determined by minimizing the elastic strain energy density $B_{pp}\left(n\right)$ with respect to orientation n [39]:

(20) $$\frac{\partial B_{pp}\left(n\right)}{\partial n}=0$$

where *p* represents the *p*^th^ variant of martensite phase. According to Equation (20), the minimum $B_{pp}\left(n\right)$ can be obtained at the habit plane normal. The results of all six variants are shown in Table 8. It can be seen that the predicted habit planes are almost the same as the predicted results from the PTMT. The angular deviation of the habit plane from KS theory is ~2.1° with respect to the observed {3 3 4} plane. However, as analyzed by Gao [16], such a habit plane in Ti-6Al-4V alloy is not an invariant habit plane, because the minimum $B_{pp}\left(n\right)$ is larger than 0. In the PTMT calculation, the eigenvalue of $U_{i}^{2}$ that is closest to 1 is 1.0034, which indicates that the predicted habit plane between a single variant and matrix is not an invariant plane. However, it is very close to 1, thus, the twinned regions of two variants with small volume fraction of one twinned variant exist in the microstructure. Moreover, different dislocations and structural ledges have been observed in the interface between α variant and β matrix [29,31], which produce a semi-coherent zigzag interface. Therefore, the generally accepted {3 3 4} habit planes only refer to a semi-coherent interface, which would be the main source of the small angular deviation for the PTMT and micro-elasticity theory calculation.

The habit plane separating the twinned region and the β matrix can be determined in a similar way but the transformation strain with two twinned variants for Equation (16) is:

(21) $$\varepsilon_{ij}=\lambda \varepsilon_{ij}\left(p\right)+\left(1-\lambda \right)\varepsilon_{ij}\left(q\right)$$

The results for twinned variants with combination of 1/2, 1/3, 1/4, 1/5 and 1/6 are listed in Table 9. From Table 9 it can be seen that the predicted habit normal from twinned variants have larger angular deviation than the habit plane from a single variant. But minimum $B_{pq}$ is much smaller than the one of a single variant, except for the $B_{12}$ between variants 1 and 2. During martensitic transformation, the microstructure evolution is dominated by the elastic strain energy, which can be reduced by twins and dislocation glide. In the present work dislocations at the interface are ignored and, thus, the twinned region relaxes the larger part of the elastic strain energy, which becomes obvious by comparing the $B_{min}\left(n\right)$ between twinned and single variants. However, from PTMT, one of the twinned variants has a very small volume fraction and is hardly observed by experiments. Shi et al. [46] calculated the minimum $B_{pp}\left(n\right)$ with consideration of dislocations at different faces in the interfacial region between α variants and β matrix, and predicted the habit plane with ~0.8° angular deviation with respect to experiments, while Cayron [54] concluded that the atomic shuffle is necessary for BCC to HCP phase transformation. Therefore, it can be concluded that, even if the twinned region can reduce the elastic strain energy to a large extent, the dislocation would have a larger effect on the microstructure evolution for all 12 crystallographic variants during martensitic transformation. For the twinned region with variant 1 and 2, the type of twin is compound. Bhattacharya [12] implied that there is no invariant plane in compound twins. The minimum $B_{12}\left(n\right)$ with compound twinned variant 1 and 2 is about 10 times larger than for a single variant, which would explain that no invariant plane exists for compound twins.

### 5.2. Crossing Twins

As reported in B2 to B19/B19′ martensitic transformation for TiNiPt alloy [15], crossing twins can be formed with 4 different variants. 6 solutions can be obtained based on Equation (9) for Ti-6Al-4V alloy with combinations of variants 1/2/3/4, 1/2/5/6 and 3/4/5/6. Similar to the β-Ti alloy [17], a crossing structure can be classified into C-II and I-C types for each variant combination in the current work.

**Figure 5:**
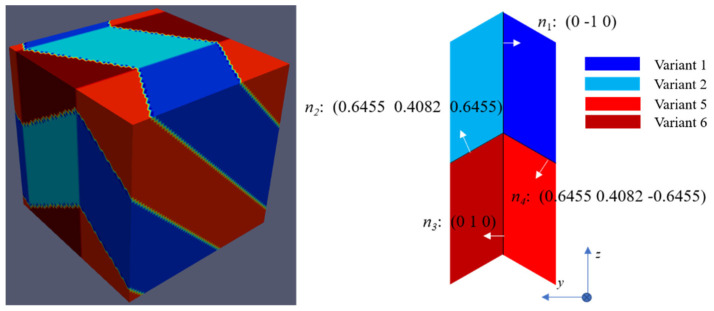
Crossing twinned microstructure with variants 1/2/5/6 and their corresponding shear vectors in the interfacial regions.

The solutions of C-II crossing twins with variant 1/2/5/6 are taken here as an example, as illustrated in Figure 5. By taking $U_{i}\;$ = $U_{1}$; $U_{j}$ = $U_{2}$; $U_{k}$ = $U_{6}$ and $U_{l}$ = $U_{5}$, the 4 invariant plane normal directions are $n_{1}$ = (0 −1 0); $n_{2}$ = (0.6455 0.4082 0.6455); $n_{3}$ = (0 1 0) and $n_{4}$= (0.6455 0.4082 −0.6455). A small angular deviation of ~1.1° of $n_{1}n_{2}n_{3}n_{4}$ from the identity matrix ***I*** implies that the crossing twins can be predicted geometrically and that no dislocations are involved in the interfacial region by PTMT. However, to the authors’ knowledge, no experimental work about such a special crossing twin with four transformation variants has been reported for Ti-6Al-4V alloy. A more detailed explanation based on the elastic energy will be given in the next section.

### 5.3. Triangular Morphology within 3 Variants

**Figure 6:**
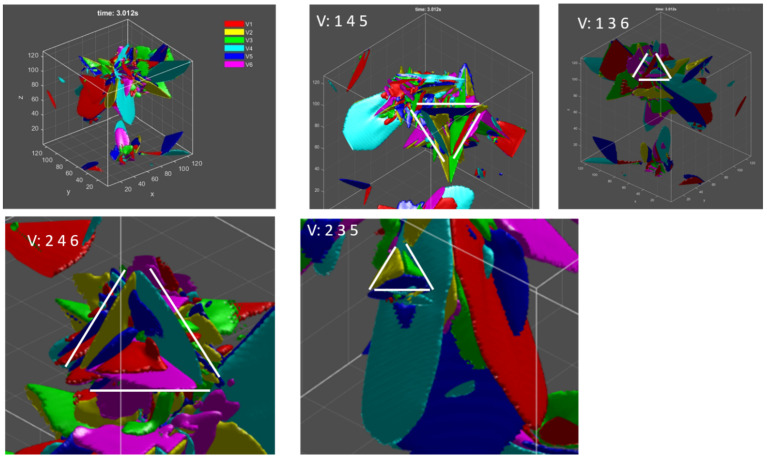
Triangular morphology with different variant combinations during martensitic transformation by phase field modelling.

The triangular morphology or pyramid structures are commonly observed within many alloys during martensitic transformation, which is also reproduced by the current phase field model. Four different variants clusters are presented in Figure 6. This type of structure, formed on the basis of reduction of elastic strain energy, is known as a self-accommodation phenomenon. This nearly pyramidal structures cannot be explained geometrically based on the PTMT, but Gao [16] proposed a similar geometrical method to explain it, which will be employed here.

Unlike the crossing twins, whereby a quadruple connection point has to be formed between four variants and all shear vectors have to be in a plane, the three variants within a pyramidal structure needs to form at an interfacial plane between them, which can be expressed geometrically as:

(22a) $$R_{ji}U_{j}-U_{i}=a_{1}⊗n_{1};\;$$

(22b) $$R_{kj}U_{k}-U_{j}=a_{2}⊗n_{2};\;$$

(22c) $$R_{ik}U_{i}-U_{k}=a_{3}⊗n_{3};\;$$

For $C_{6}^{3}$ = 20 combinations of 3-variant clusters, the minimum misorientation angle of $n_{1}n_{2}n_{3}$ from the identity matrix ***I*** is ~ 7.01°, and solutions of three variants that form the clusters of Figure 6 are consistent with the phase field simulation, i.e., the formation of 3-variant clusters obtained by phase field modelling with elastic strain energetic view can be explained geometrically with the compatibility condition of Equation (22). However, if any variant in the 3-variant clusters is replaced by the β matrix, i.e., assuming $U_{k}$= ***I*** in Equation (22), the minimum misorientation angle of $n_{1}n_{2}n_{3}$ from the identity matrix ***I*** is ~ 2.41°, which is smaller than the angular deviation of 3-variants.

The small angular deviation of 2/3-variant clusters would not explain geometrically the formation of triangular morphology with three variants. However, by considering the degree of self-accommodation (DSA) in Ref. [58], it can be explained based on elastic strain energy minimization. By setting the transformation matrix in Equation (3) as input, the transformation matrix of the *n*-variant clusters can be given as:

(23) $$U^{\left(n\right)}=\sum_{i=1}^{n}U_{i}$$

and then the transformation strain for the *n*-variant cluster is obtained according to Equation (10). The DSA is derived according to the following equation by considering the Von Mises equivalent strain and stress, and the elastic strain energy density $W^{\left(n\right)}$ of the *n*-variant clusters:

(24) $$\mathrm{DSA}=\frac{W^{\left(1\right)}-W^{\left(n\right)}}{W^{\left(1\right)}}*100\%$$

Given different combinations of 2-, 3- and 4-variant clusters, which are assumed to correspond to the patterns of 2 variants with matrix, triangular morphology and crossing twins, respectively, it can be concluded that the clusters with the variants exhibited in Figure 6 are mostly favored during martensitic transformation.

About the crossing twins mentioned in the previous section, these have been observed and predicted by experiments and PTMT for TiNi alloy [16,70]. However, in the currently studied Ti-6Al-4V alloy, such crossing twin morphology can be predicted geometrically but has not been reported thus far. The DSA for the four-variant clusters is higher than for the three-variant cluster in TiNi alloy, but smaller in the Ti-6Al-4V alloy, which would explain why few reports about crossing twins in Ti-6Al-4V alloy are reported. The occurrence of simulated crossing twins in Figure 5 is due to the fact that only four variants that can form such a special microstructure are taken into consideration, but any three of them cannot evolve into a triangular morphology. In reality, there are six correspondence variants and twelve crystallographic variants. The absence and presence of some variants lead to the change of interaction energy, thus decomposing the formation of special crossing twins in reality.

## 6. Summary

The Phenomenological Theory of Martensitic Transformation (PTMT) is a geometrical theory with specific lattice parameters while the Phase Field Model (PFM) is based on the reduction of Gibbs free energy in the simulated system. In terms of crystallographic features during martensitic transformation for Ti-6Al-4V alloy both theories are capable of predicting the habit plane normal with satisfactory results. The PTMT and PFM reveal different aspects with regard to microstructural study. They can be compared on the following points:

- The PTMT is a geometrical (mechanical) theory requiring less time-consuming calculations while the PFM is based on thermodynamic principles; the PFM simulations are computationally more expensive;
- The PTMT is able to calculate the volume fractions of twinned regions and twinning elements, predict the orientation relationship between the parent and product phase, which the PFM in its current implementation is not capable of;
- The PTMT is not able to simulate the microstructural evolution as a function of time, but the PFM has this ability to capture the event of nucleation and growth of α variants and reveal the formation of triangular morphology of variant clusters;
- The special crossing twins observed in other alloys can be predicted geometrically by the PTMT; based on the elastic strain energy minimization with PFMs it was revealed that such special twin configurations are not compatible with the occurrence and/or absence of some variants.

Based on the results and analysis, with proper calculation parameters as input and acceptable angular deviation (normally less than 5°), the present numerical works about PTMT and microstructure simulation from phase field models can be applied to other alloys.

## Figures and Tables

**Table 1:** Predicted habit plane normal and shear vector from Equation (4) in the parent phase reference system.

Variant	Shear Vector b	Habit Plane Normal m
1	(0.1567 −0.0885 0.0885)	(−0.7147 −0.4946 0.4946)
	(0.1567 0.0885 −0.0885)	(−0.7147 0.4946 −0.4946)
2	(0.1567 0.0885 0.0885)	(−0.7147 0.4946 0.4946)
	(0.1567 −0.0885 −0.0885)	(−0.7147 −0.4946 −0.4946)
3	(−0.0885 0.1567 0.0885)	(−0.4946 −0.7147 0.4946)
	(0.0885 0.1567 −0.0885)	(0.4946 −0.7147 −0.4946)
4	(0.0885 −0.1567 0.0885)	(0.4946 0.7147 0.4946)
	(−0.0885 −0.1567 −0.0885)	(−0.4946 0.7147 −0.4946)
5	(−0.0885 0.0885 0.1567)	(−0.4946 0.4946 −0.7147)
	(0.0885 −0.0885 0.1567)	(0.4946 −0.4946 −0.7147)
6	(0.0885 0.0885 0.1567)	(0.4946 0.4946 −0.7147)
	(−0.0885 −0.0885 0.1567)	(−0.4946 −0.4946 −0.7147)

**Table 2:** Twin elements for compound twins with different variant combinations.

Compound	Twin Shear *a*	Twin Plane Normal *n*
Variant: 1/2	(0 −0.1824 −0.008)	(0 0 1)
	(0 −0.008 −0.1824)	(0 1 0)
Variant: 3/4	(−0.1824 0 −0.008)	(0 0 1)
	(−0.008 0 −0.1824)	(1 0 0)
Variant: 5/6	(−0.1824 −0.008 0)	(0 1 0)
	(−0.008 −0.1824 0)	(1 0 0)

**Table 3:** Twin elements for type I twins with different variant combinations.

Type I	Twin Shear *a*	Twin Plane Normal *n*
Variant:1/3	(−0.2357 −0.1968 0.1189)	(1 −1 0)
Variant:1/4	(−0.2357 0.1968 −0.1189)	(1 1 0)
Variant:1/5	(−0.2357 0.1189 −0.1968)	(1 0 −1)
Variant:1/6	(−0.2357 −0.1189 0.1968)	(1 0 1)
Variant:2/3	(−0.2357 0.1968 0.1189)	(1 1 0)
Variant:2/4	(−0.2357 −0.1968 −0.1189)	(1 −1 0)
Variant:2/5	(−0.2357 0.1189 0.1968)	(1 0 1)
Variant:2/6	(−0.2357 −0.1189 −0.1968)	(1 0 −1)
Variant:3/5	(−0.1189 0.2357 0.1968)	(0 −1 1)
Variant:3/6	(−0.1189 −0.2357 0.1968)	(0 1 1)
Variant:4/5	(0.1189 −0.2357 0.1968)	(0 1 1)
Variant:4/6	(0.1189 0.2357 0.1968)	(0 −1 1)

**Table 4:** Twin elements for type II twins with different variant combinations.

Type II	Twin Shear *a*	Twin Plane Normal *n*
Variant:1/3	(−0.2590 0.2207 0.0113)	(0.6455 0.6455 −0.4082)
Variant:1/4	(−0.2590 −0.2207 −0.0113)	(0.6455 −0.6455 0.4082)
Variant:1/5	(−0.2590 0.0113 0.2207)	(0.6455 −0.4082 0.6455)
Variant:1/6	(−0.2590 −0.0113 −0.2207)	(0.6455 0.4082 −0.6455)
Variant:2/3	(−0.2590 −0.2207 0.0113)	(0.6455 −0.6455 −0.4082)
Variant:2/4	(−0.2590 0.2207 −0.0113)	(0.6455 0.6455 0.4082)
Variant:2/5	(−0.2590 0.0113 −0.2207)	(0.6455 −0.4082 −0.6455)
Variant:2/6	(−0.2590 −0.0113 0.2207)	(0.6455 0.4082 0.6455)
Variant:3/5	(−0.0113 0.2590 −0.2207)	(0.4082 −0.6455 −0.6455)
Variant:3/6	(−0.0113 −0.2590 −0.2207)	(0.4082 0.6455 −0.6455)
Variant:4/5	(0.0113 −0.2590 −0.2207)	(−0.4082 0.6455 −0.6455)
Variant:4/6	(0.0113 0.2590 −0.2207)	(−0.4082 −0.6455 −0.6455)

**Table 5:** Predicted variant volume fraction, habit plane normal and angular deviation from experimental result [20,58].

Variants: 2/5	Volume Fraction λ of V2	Habit Plane Normal *m*	Angular Deviation (°) from {3 3 4}
Type I	0.0398	(0.4720 −0.5039 −0.7233)	3.2961
	0.0398	(−0.4771 0.5210 −0.7078)	2.5103
	0.9602	(−0.7233 0.5039 0.4720)	3.2961
	0.9602	(−0.7078 −0.5210 −0.4771)	2.5103
Type II	0.0539	(0.4720 −0.5039 −0.7233)	3.8003
	0.0539	(−0.4771 0.5210 −0.7078)	2.8937
	0.9461	(−0.7233 0.5039 0.4720)	2.8937
	0.9461	(−0.7078 −0.5210 −0.4771)	3.8003

**Table 6:** Predicted orientation relationships of different variants based on PTMT.

Variant	Predicted (0 0 0 1) Plane	$\mathrm{Predicted}\;(1\;0\;\bar{1}\;0)\;\mathrm{Plane}$	$\mathrm{Predicted}\;[1\;1\;\bar{2}\;0]$ Direction
1	${\left(0\;\bar{1}\;1\right)}_{\beta }$5.1311°	${\left(0\;1\;1\right)}_{\beta }$0.0715°	${\left[\bar{1}\;1\;1\right]}_{\beta }5.6713{}^{\circ}$
2	${\left(0\;\bar{1}\;\bar{1}\right)}_{\beta }$5.1311°	${\left(0\;\bar{1}\;1\right)}_{\beta }$0.0715°	${\left[\bar{1}\;\bar{1}\;1\right]}_{\beta }5.6713{}^{\circ}$
3	${\left(\bar{1}\;0\;1\right)}_{\beta }$4.6233°	${\left(1\;0\;1\right)}_{\beta }0.4025{}^{\circ}$	${\left[1\;1\;1\right]}_{\beta }$5.4979°
4	${\left(\bar{1}\;0\;\bar{1}\right)}_{\beta }$5.7329°	${\left(\bar{1}\;0\;1\right)}_{\beta }$0.0715°	${\left[\bar{1}\;1\;1\right]}_{\beta }$0.9657°
5	${\left(\bar{1}\;1\;0\right)}_{\beta }4.6233{}^{\circ}$	${\left(\bar{1\;}\bar{1}\;0\right)}_{\beta }2.2665{}^{\circ}$	${\left[\bar{1}\;\bar{\;1}\;1\right]}_{\beta }5.4979{}^{\circ}$
6	${\left(\bar{1}\;\bar{1}\;0\right)}_{\beta }4.6233{}^{\circ}$	${\left(1\;\bar{1}\;0\right)}_{\beta }2.2665{}^{\circ}$	${\left[1\bar{\;1}\;1\right]}_{\beta }0.9657{}^{\circ}$

**Table 7:** Simulation parameters used in the phase field simulation.

Physical Parameters	Symbol	Value	Unit
Temperature	T	1073	K
Grid size	$d_{x}$	25	nm
System size	$l_{x}$ $,\;l_{y}$ $,\;l_{z}$	128, 128, 128	-
Interface thickness	δ	$5d_{x}$	nm
Interfacial energy	γ	$50\times 10^{-3}$	$J/m^{2}$
Molar volume	$V_{m}$	$10^{-5}$	$m^{3}/\mathrm{mol}$
Kinetic coefficient	M	$1.6\times 10^{-7}$	$J/m^{3}/s$
Elastic constant	$C_{11}$ $,\;C_{12}$ $,\;C_{44}$	97.7, 82.7, 7.5	GPa
Time step	*dt*	$2\times 10^{-3}$	s
Normalization factor	ΔM	$10^{-18}$	$m^{2}\;\mathrm{mol}/J/s$

**Table 8:** Predicted habit plane normal, minimum $B_{pp}\left(n\right)$ and angular deviation.

Variant	Habit Plane Normal n	$B_{min}\left(n\right)$ $\;(J/m^{3})$	Angular Deviation(°) from {3 3 4}
1	(0.7122 −0.4972 0.4955)	1.70 × 10^5^	2.10
2	(0.7122 −0.4972 −0.4955)	1.70 × 10^5^	2.10
3	(−0.4972 0.7122 0.4955)	1.70 × 10^5^	2.10
4	(−0.4972 0.7122 −0.4955)	1.70 × 10^5^	2.10
5	(−0.4968 0.4968 0.7115)	1.75 × 10^5^	2.04
6	(0.4968 0.4968 0.7115)	1.75 × 10^5^	2.04

**Table 9:** Predicted habit plane normal, minimum $B_{pq}\left(n\right)$ and angular deviation with twinned variants.

Variant	Habit Plane Normal n	$B_{min}\left(n\right)$ $\;(J/m^{3})$	Angular Deviation (°) from {3 3 4}
1/2	(−0.7141 0.4919 0.4982)	1.65 × 10^6^	2.27
1/3	(0.4811 0.7176 −0.5036)	2.13 × 10^4^	2.71
1/4	(0.4811 −0.7176 0.5036)	2.13 × 10^4^	2.71
1/5	(−0.4742 0.5245 0.7071)	2.10 × 10^4^	2.71
1/6	(0.4742 0.5245 0.7071)	2.10 × 10^4^	2.71

## Data Availability

The program of phase field modelling can be found through the link (https://github.com/hxiang1991/phase-field-simulation-of-Ti-6Al-4V).

## Supplementary Materials

The following supporting information can be downloaded at: https://www.mdpi.com/article/10.3390/ma15155325/s1, Video: microstructure evolution with 6 correspondence variants.
